# Crucial Role for Lipoteichoic Acid Assembly in the Metabolic Versatility and Antibiotic Resistance of Staphylococcus aureus

**DOI:** 10.1128/iai.00550-22

**Published:** 2023-06-22

**Authors:** Troy A. Burtchett, John C. Shook, Laura E. Hesse, Philip C. Delekta, Robert S. Brzozowski, Alhakam Nouri, Alexa J. Calas, Catherine M. Spanoudis, Prahathees J. Eswara, Neal D. Hammer

**Affiliations:** a Department of Microbiology and Molecular Genetics, Michigan State University, East Lansing, Michigan, USA; b Department of Cell Biology, Microbiology, and Molecular Biology, University of South Florida, Tampa, Florida, USA; University of Illinois Chicago

**Keywords:** *Staphylococcus aureus*, lipoteichoic acid, metabolism, small-colony variant, membrane potential, glycolipid anchor, *ypfP*, ion homeostasis, cation

## Abstract

Staphylococcus aureus is a public health threat due to the prevalence of antibiotic resistance and the capacity of this organism to infect numerous organs in vertebrates. To generate energy needed to proliferate within tissues, S. aureus transitions between aerobic respiration and fermentation. Fermentation results in a distinct colony morphology called the small-colony variant (SCV) due to decreased membrane potential and ATP production. These traits promote increased resistance to aminoglycoside antibiotics. Consequently, SCVs are associated with persistent infections. We hypothesize that dedicated physiological pathways support fermentative growth of S. aureus that represent potential targets for treatment of resistant infections. Lipoteichoic acid (LTA) is an essential component of the Gram-positive cell envelope that functions to maintain ion homeostasis, resist osmotic stress, and regulate autolytic activity. Previous studies revealed that perturbation of LTA reduces viability of metabolically restricted S. aureus, but the mechanism by which LTA supports S. aureus metabolic versatility is unknown. Though LTA is essential, the enzyme that synthesizes the modified lipid anchor, YpfP, is dispensable. However, *ypfP* mutants produce altered LTA, leading to elongation of the polymer and decreased cell association. We demonstrate that viability of *ypfP* mutants is significantly reduced upon environmental and genetic induction of fermentation. This anaerobic viability defect correlates with decreased membrane potential and is restored upon cation supplementation. Additionally, *ypfP* suppressor mutants exhibiting restored anaerobic viability harbor compensatory mutations in the LTA biosynthetic pathway that restore membrane potential. Overall, these results demonstrate that LTA maintains membrane potential during fermentative proliferation and promotes S. aureus metabolic versatility.

## INTRODUCTION

Staphylococcus aureus poses a considerable threat to public health due its capacity to rapidly develop resistance to antibiotics and infect numerous organs in vertebrates. The latter is underscored by the fact that S. aureus is the leading cause of skin and soft tissue infections, endocarditis, and osteomyelitis (1–3). Additionally, S. aureus is also prevalent in the lungs of people afflicted with cystic fibrosis (CF) (4, 5). CF patients are often colonized with Pseudomonas aeruginosa and receive multiple rounds of aminoglycoside antibiotics to treat bacterial infection (6). In response to both of these environmental challenges, S. aureus develops inactivating mutations in pathways that support aerobic respiration, such as heme synthesis (7–9). Consequently, heme auxotrophs can be isolated from CF patients (10). In addition to enhanced resistance to aminoglycosides, genetic inactivation of heme synthesis results in a distinct colony morphology referred to as the small-colony variant (SCV). SCVs rely exclusively on fermentation to proliferate, as aerobic respiration is inhibited in these cells. Impaired respiration leads to reduced proton motive force (PMF), which explains enhanced SCV aminoglycoside resistance, as import of the antibiotic is dependent upon the PMF (11). PMF comprises the proton gradient (ΔpH) and membrane potential (Δψ). We provide evidence that ion homeostasis mediated by the essential cell envelope polymer lipoteichoic acid (LTA) is a key factor in maintaining the membrane potential in S. aureus cells in which respiration is arrested.

The staphylococcal cell envelope contains two teichoic acid polymers, LTA and wall teichoic acid (WTA), which together make up a “continuum of anionic charge” (12–14). S. aureus synthesizes type 1 LTA, consisting of a glycolipid anchor, diglucosyl-diacylglycerol (Glc_2_-DAG), to which a chain of 1,3-linked glycerolphosphates (GroP) is attached (15, 16). Synthesis of the Glc_2_-DAG glycolipid anchor is catalyzed by the enzyme YpfP, which uses UDP-glucose as a substrate to covalently link two glucose moieties to diacylglycerol present on the inner leaflet of the plasma membrane (17–19). Glc_2_-DAG is flipped to the exterior leaflet of the plasma membrane by LtaA, which is cotranscribed with *ypfP*. LtaS transfers GroP moieties from phosphatidylglycerol to Glc_2_-DAG, creating mature LTA (20). Despite the fact that LTA is indispensable, LtaS is the only essential enzyme in the pathway, indicating that this protein is a potential target for therapeutic intervention (21). Conversely, *ltaA* and *ypfP* mutants are viable, but both mutant strains produce altered LTA. In some strain backgrounds inactivation of *ypfP* reduces cell-associated LTA by nearly 90% (18).

A molecular explanation for the essentiality of LTA has been elusive, as LTA is involved in numerous physiological processes, such as ion homeostasis and regulation of autolysin (18, 19, 22). Neutralizing the negative charge of LTA GroP via addition of d-alanine allows S. aureus to resist cationic antimicrobial peptides (23). Cells that conditionally lack LTA display extreme morphological defects (20). Previous reports showed that perturbing the metabolic potential of some S. aureus strains in which LTA or WTA production is impeded reduces viability, and it has been postulated that LTA-mediated ion homeostasis contributes to the PMF, but direct evidence has not been presented (19, 24, 25). We build on these studies to show that inactivation of *ypfP* impairs proliferation of cells in which respiration is arrested. The *ypfP* mutant with arrested respiration exhibits reduced membrane potential and reduced viability. The anaerobic proliferation defects are suppressed upon supplementation with the alternative electron acceptor KNO_3_ or cations. Additionally, *ypfP* suppressor mutants have restored respiration-arrested viability and membrane potential. Together, these results support the conclusion that the LTA glycolipid anchor facilitates maintenance of the membrane potential under respiration-arresting, fermentative growth conditions.

## RESULTS

### Respiration arrest impairs proliferation of S. aureus *ypfP* mutants.

**FIG 1:**
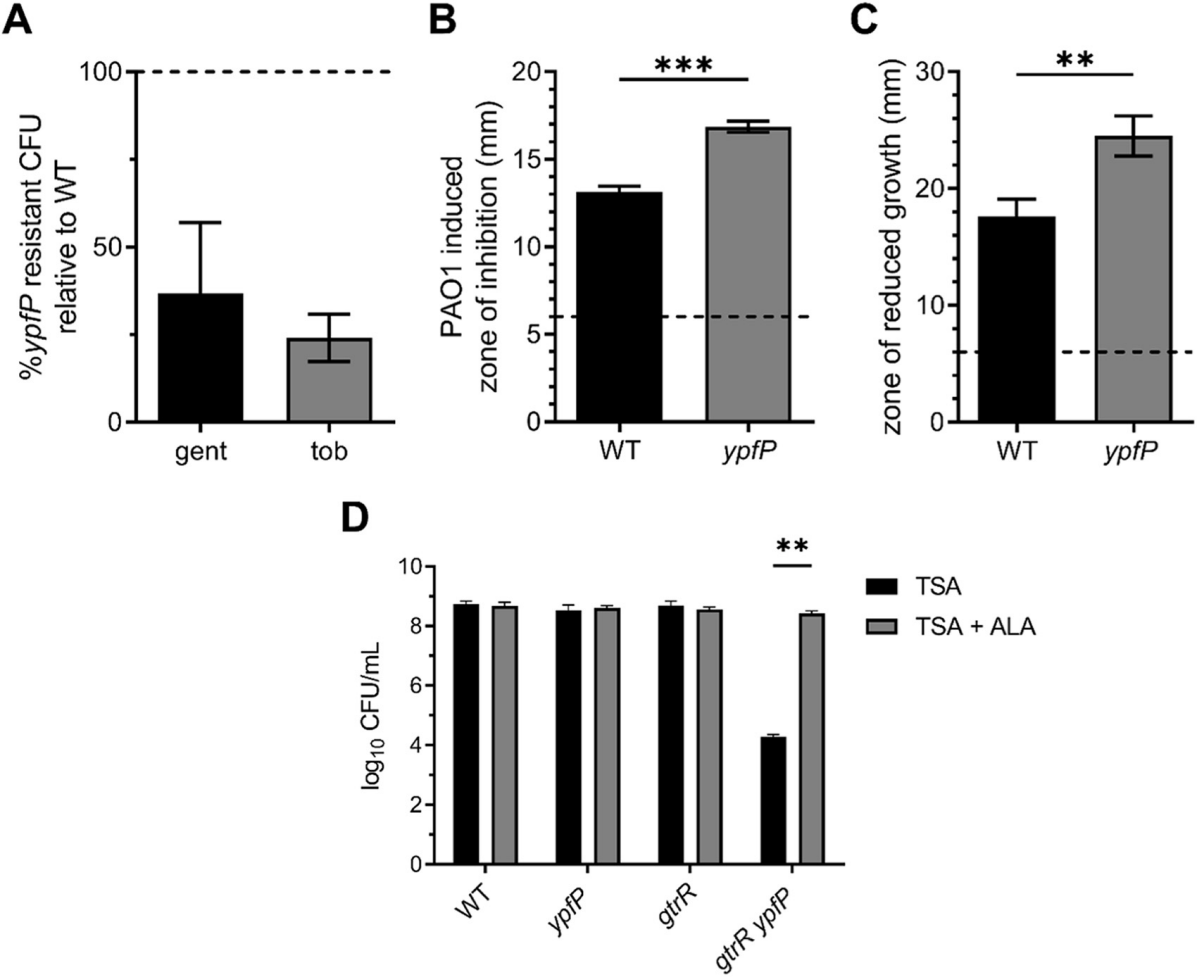
Respiration-arresting conditions impair S. aureus *ypfP* proliferation. (A) CFU of WT or *ypfP* mutant cells grown on TSA supplemented with 5 μg mL^−1^ of gentamicin (gent) or tobramycin (tob) were enumerated after 24 h at 37°C. Values are percentages of the WT value, where the number of CFU of the *ypfP* mutant was divided by the number of CFU of the WT and the WT value was set to 100% (dotted line). Data are means from at least five independent experiments. Error bars represent one standard deviation from the mean. (B) Zones of inhibited growth generated by colonies of P. aeruginosa spotted on top of S. aureus WT or *ypfP* mutant lawns were measured after 24 h at 37°C. Data are means from three independent experiments performed in triplicate. Statistical significance was determined by an unpaired two-tailed *t* test for unequal variance. *P < *0.0001. Error bars represent one standard deviation from the mean. (C) The zone of reduced growth generated by HQNO on lawns of WT or *ypfP* mutant cells was measured after 24 h at 37°C. Data are means from three independent experiments performed in triplicate. Error bars represent one standard deviation from the mean. Statistical significance was determined by an unpaired two-tailed *t* test for unequal variance. *P < *0.01. (D) CFU of WT and *ypfP*, *gtrR* or *gtrR ypfP* mutant strains were quantified after 24 h (for respiring colonies) or 48 h (for respiration arrest colonies) of growth on TSA or TSA supplemented with ALA at 37°C. Error bars represent one standard deviation for three independent experiments. Statistical significance was determined by two-way analysis of variance (ANOVA) with a Šidák method for multiple comparisons. **, *P* < 0.01.

S. aureus arrests respiration upon exposure to aminoglycoside antibiotics, resulting in the development of resistant colonies (7, 26, 27). To assess LTA contributions to aminoglycoside resistance, we challenged S. aureus with 5 μg mL^−1^ of the clinically relevant aminoglycosides gentamicin and tobramycin. Compared to the number of aminoglycoside-resistant colonies generated in the corresponding wild-type (WT) strains, the *ypfP* mutant produced considerably fewer resistant colonies (~63% and ~76% decrease for gentamicin and tobramycin, respectively) (Fig. 1A). To further investigate the aminoglycoside sensitivity, we monitored the morphology of WT and *ypfP* mutant cells in response to gentamicin via fluorescence microscopy (see Fig. S1 in the supplemental material). The untreated WT and *ypfP* mutant appear similar, as indicated by membrane staining (FM4-64, red). While gentamicin treatment caused abnormal membrane clumping in the WT (46%; *n* = 100), it was more pronounced in *ypfP* mutant cells (57%; *n* = 100). Increased membrane clumping, possibly due to improper membrane invagination or loss of membrane integrity, is an indication that cells are *en route* to lysis (28) and provides a visual depiction of the increased gentamicin susceptibility of *ypfP* mutant cells (Fig. S1).

Lungs of CF patients are often colonized with S. aureus and the Gram-negative, opportunistic pathogen Pseudomonas aeruginosa. Studies have shown that P. aeruginosa induces respiration arrest and development of SCVs in S. aureus via production of 2-heptyl-4-hydroxyquinolone *N*-oxide (HQNO) and pyocyanin (29–33). As coculture with P. aeruginosa induces S. aureus respiration arrest and the *ypfP* mutant demonstrates impaired capacity to resist aminoglycosides, we reasoned that the *ypfP* mutant would be more susceptible to P. aeruginosa, HQNO, and/or pyocyanin. To test this, a lawn of WT S. aureus or the *ypfP* mutant was plated onto tryptic soy agar (TSA) and P. aeruginosa PAO1 was spotted on top (33). The zone of inhibition induced by P. aeruginosa was quantified. Compared to WT zones of inhibition (~13 mm), the *ypfP* mutant demonstrates a larger area of inhibited growth (~17 mm) (Fig. 1B). We hypothesized that the increased sensitivity of the *ypfP* mutant is due to the respiration-inhibiting effects of HQNO or pyocyanin and used a Kirby-Bauer disk diffusion assay with HQNO or pyocyanin to test this. Compared to WT cells, the *ypfP* mutant demonstrated enhanced susceptibility to HQNO but not pyocyanin (Fig. 1C and data not shown). In total, these findings demonstrate that genetic inactivation of *ypfP* sensitizes S. aureus to respiration arrest induced by P. aeruginosa coculture and exposure to aminoglycosides.

A limitation of using small molecules or competition with other organisms to induce respiration arrest is the potential for off-target effects. To further resolve the role of the LTA glycolipid anchor in S. aureus metabolic versatility, we genetically inactivated *ypfP* in a heme synthesis SCV mutant, a Δ*gtrR* (formerly *hemA*) mutant, which has been previously used as a model SCV in S. aureus (8). Heme synthesis and respiration is restored in the Δ*gtrR* mutant upon supplementation with the heme precursor δ-aminolevulinic acid (ALA) (8). This allows the Δ*gtrR* cells to perform continuous aerobic respiration as the strain undergoes additional genetic manipulation. Subsequent phenotypic analysis of the resulting double mutant under respiration-arresting conditions is achieved by simply culturing the cells in medium devoid of ALA. Plating the Δ*gtrR ypfP* double mutant on solid medium lacking ALA results in an approximately 4-log decrease in CFU compared to WT or Δ*gtrR ypfP* mutant cells plated on medium supplemented with ALA (Fig. 1D). In total, these results imply that inactivation of *ypfP*, which lacks the LTA glycolipid anchor and reduces cell-associated LTA, impairs proliferation of S. aureus during respiration arrest (17–19).

### Anaerobiosis reduces *ypfP* mutant viability.

**FIG 2:**
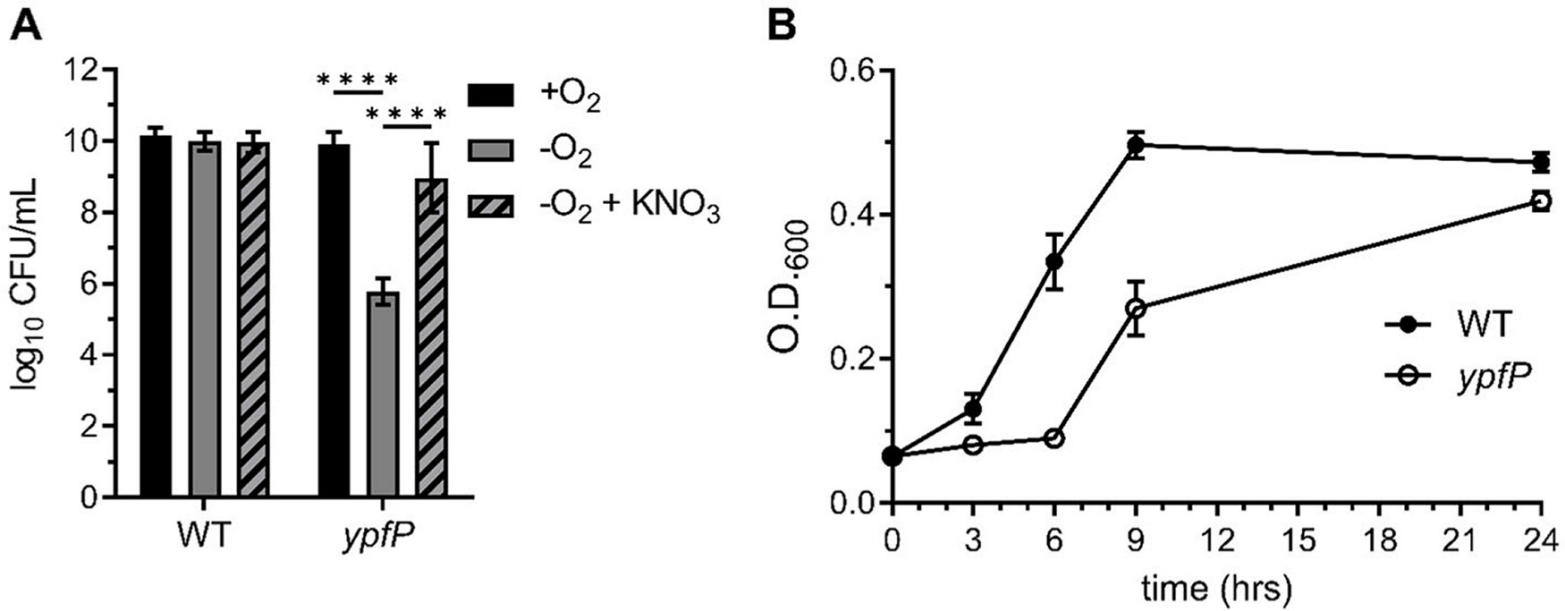
*ypfP* mutant cells demonstrate reduced anaerobic proliferation. (A) CFU of WT or *ypfP* mutant bacteria generated after incubation under aerobic (+O_2_) or anaerobic (−O_2_) conditions were enumerated after 24 h at 37°C. Potassium nitrate (KNO_3_; 100 mM) was used to induced anaerobic respiration. Data are means from three independent experiments performed in triplicate. Error bars represent one standard deviation from the mean. Statistical significance was determined by a two-way ANOVA with Tukey’s multiple-comparison test. ****, *P < *0.0001. (B) The WT and the *ypfP* mutant were subcultured 1:100 from an overnight culture and grown anaerobically at 37°C. Growth was measured at the indicated time points by monitoring OD_600_. The experiment was performed in triplicate. Error bars represent standard deviations from the mean.

We reasoned that the viability defect of the Δ*gtrR ypfP* mutant could be attributed exclusively to respiration arrest or to a combination of respiration arrest and increased oxidation. To distinguish between these possibilities, we enumerated CFU generated by the *ypfP* mutant cultured under aerobic and anaerobic conditions. The *ypfP* mutant cells cultured anaerobically demonstrated a 4-log reduction in viability (Fig. 2A); a similar decrease was observed when respiration was arrested in a Δ*gtrR ypfP* double mutant (Fig. 1D). Supplementation of the anaerobic cultures with the alternative electron acceptor KNO_3_ rescues *ypfP* mutant viability, supporting the conclusion that reduced viability results from decreased respiration (Fig. 2A). The *ypfP* mutant cells also exhibit a significant lag phase compared to the WT when cultured in anaerobic conditions, although the mutant cells ultimately achieved a WT-like terminal optical density at 600 nm (OD_600_) (Fig. 2B). A growth defect was not observed when *ypfP* cells were cultured aerobically (Fig. S2). Together, these results show that *ypfP* is needed for maximal anaerobic proliferation.

### The anaerobic viability defect is specific to inactivation of *ypfP*.

**FIG 3:**
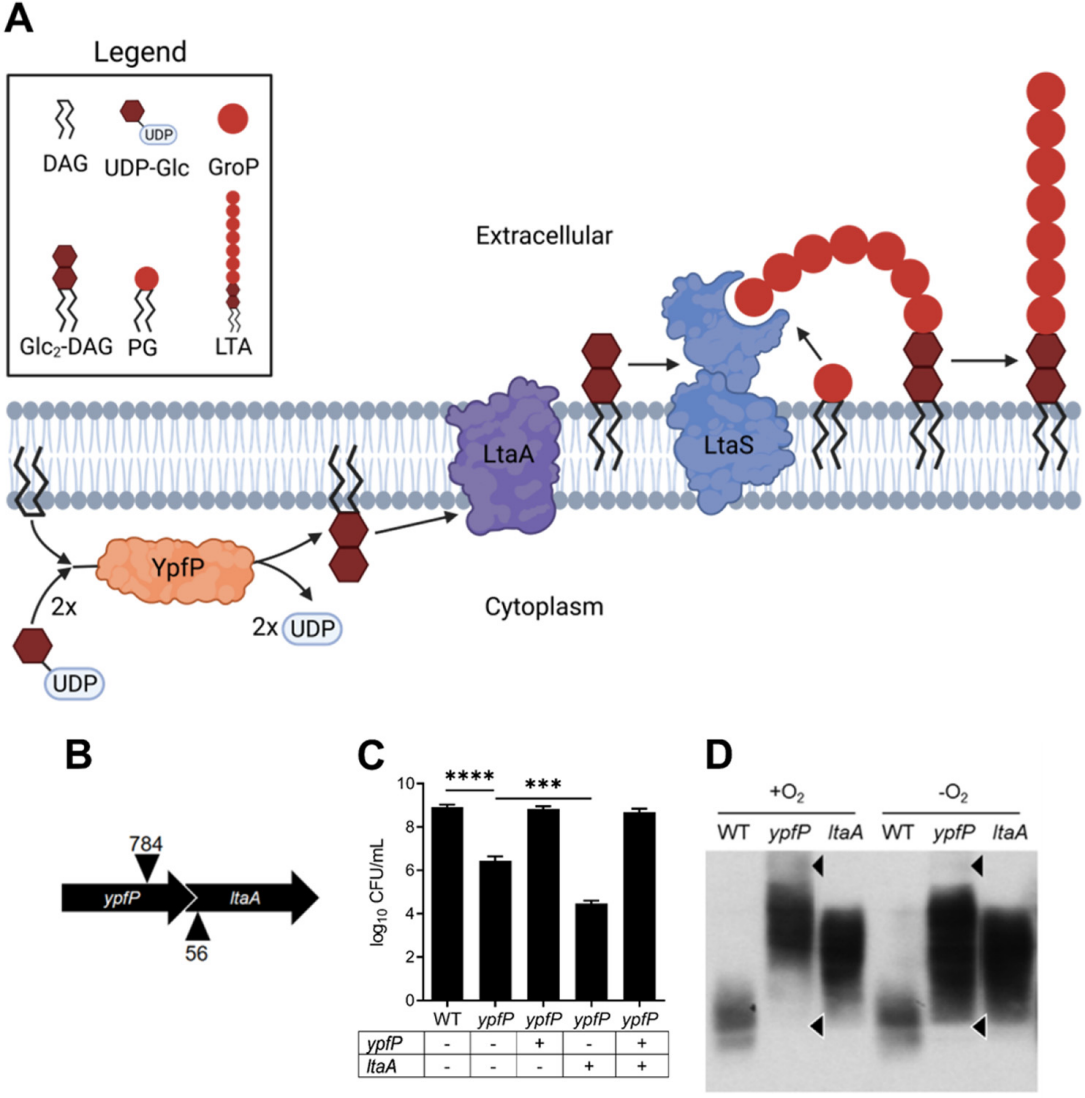
*ypfP*, not *ltaA*, is responsible for alterations in viability and LTA production under anaerobic conditions. (A) The LTA-biosynthetic pathway in S. aureus. YpfP uses DAG and two UDP glucose (UDP-Glc) molecules to generate the lipid anchor Glc_2_DAG. Glc_2_-DAG is then flipped to the outer leaflet of the membrane by LtaA, and glycerol phosphate (GroP) taken from phosphatidylglycerol (PG) is added directly to the anchor by LtaS. Illustration created using Biorender.com. (B) Illustration of the *ypfP ltaA* operon demonstrating the 41-bp *ypfP*-*ltaA* overlap. Locations of transposon insertions are indicated with arrowheads. (C) WT and *ypfP* mutant NWMN cells harboring the S. aureus expression vector pOS containing the indicated genes were spot plated on TSA and incubated in an anaerobic chamber for 24 h of growth at 37°C prior to CFU enumeration. Data are means from three independent experiments performed in triplicate. Error bars represent one standard deviation from the mean. Significance was determined via two-tailed *t* test. ****, *P* < 0.0001; ***, *P* < 0.001. (D) Representative immunoblot using a monoclonal anti-LTA antibody and a secondary antibody conjugated with horseradish peroxidase shows the altered LTA profiles of NWMN *ypfP* and *ltaA* mutants cultured under aerobic (+O_2_) and anaerobic (−O_2_) conditions compared to the WT. Differences in the LTA profile are indicated with arrowheads.

The *ypfP* mutant demonstrates reduced growth under multiple respiration-arresting conditions and an overt viability defect when cultured anaerobically. YfpP produces Glc_2_-DAG that is flipped to the outer leaflet of the cytoplasmic membrane by LtaA (Fig. 3A) (34). *ypfP* is cotranscribed with *ltaA*, and the transposon insertion used to inactivate *ypfP* is located upstream of *ltaA* (Fig. 3B). Therefore, the phenotypes we observed could be due to polar effects on *ltaA* and not specific inactivation of *ypfP* (17). To test this, complementation plasmids using the pOS P*_lgt_* vector carrying *ypfP*, *ltaA*, or *ypfP ltaA* were transformed into the *ypfP* mutant to determine the contribution of each gene to decreased anaerobic viability (35). *ypfP* mutant cells harboring a WT copy of *ypfP* rescued viability whereas *ypfP* mutant cells containing an empty vector displayed reduced anaerobic viability. Complementation of the *ypfP* mutant with *ltaA* resulted in a further reduction in anaerobic viability compared to the empty vector control (Fig. 3C). These results demonstrate that the viability defect is due to *ypfP* inactivation. Consistent with this, the *ltaA* mutant generated WT CFU when cultured anaerobically (data not shown).

*ypfP* mutants lack Glc_2_-DAG, and consequently, LTA is anchored to DAG (17, 18). Similarly, LTA is also predominantly anchored to DAG in *ltaA* mutants despite Glc_2_-DAG production in this mutant background (17). Although GroP is anchored to the same molecule in both mutants, cell-associated LTA profiles produced by these mutants are distinct (17, 18). Our results show that genetic inactivation of *ypfP*, but not *ltaA*, reduces anaerobic viability. Therefore, we reasoned that the LTA profile produced by the *ypfP* mutant in respiration-arresting environments may be altered further. To test this, we surveyed LTA produced in each mutant strain under aerobic and anaerobic conditions via immunoblotting. In addition to the previously reported distorted LTA profiles produced by aerobically cultured *ypfP* and *ltaA* mutant cells (Fig. 3D), anaerobically cultured *ypfP* mutants demonstrate further alterations to LTA underscored by loss of high-molecular-weight (HMW) species and gain of low-molecular-weight species (Fig. 3D) (17). Notably, anaerobiosis does not affect LTA production in the *ltaA* mutant, implicating altered LTA production as a contributing factor to reduced proliferation of the *ypfP* mutant during respiration arrest.

### Supplementation of the growth medium with cations rescues the anaerobic viability defect of the *ypfP* mutant.

**FIG 4:**
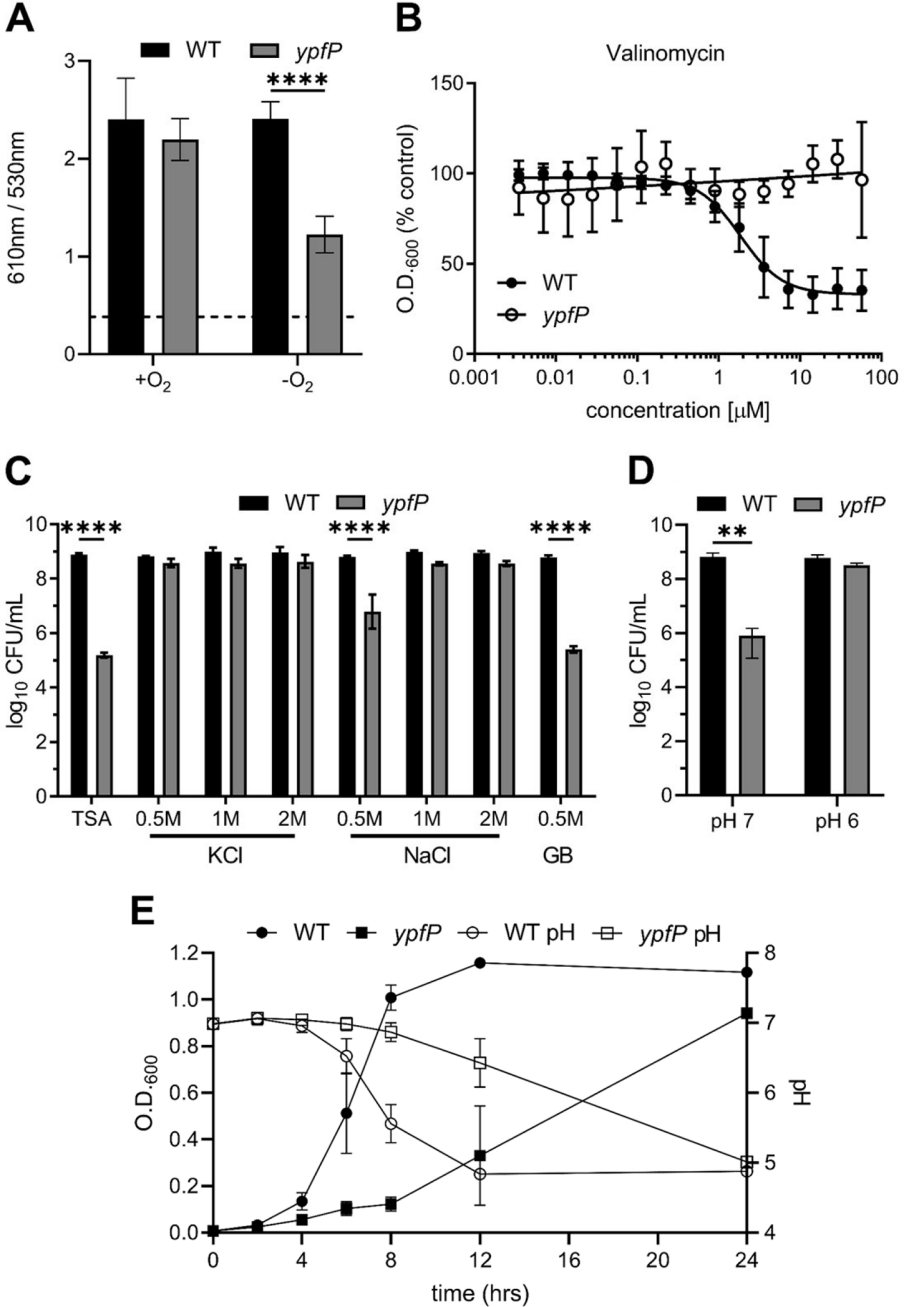
Cation supplementation restores anaerobic viability of *ypfP* mutants undergoing respiration arrest. (A) Membrane potential of the WT and *ypfP* mutant. The *y* axis shows the quotient of the 610 nm and 530 nm emission spectra. Data are means from three independently performed experiments performed in technical triplicate. Error bars represent one standard deviation from the mean. Significance was determined using two-way ANOVA. ****, *P* < 0.0001. (B) The antimicrobial activity of the potassium-specific ionophore valinomycin was determined for WT and *ypfP* mutant cells. The WT and the *ypfP* mutant were normalized to an OD_600_ of 0.5 and were allowed to grow in liquid medium in the presence of various concentrations of valinomycin anaerobically for 16 h. Cells were suspended by pipetting, and the OD_600_ was measured. Results are percent OD_600_ compared to that of the untreated control. The mean for five independent experiments performed in triplicate is shown. The regression line was mapped using GraphPad Prism. Error bars represent one standard deviation from the mean. (C) Anaerobic viability of the WT and the *ypfP* mutant on medium supplemented with various concentrations of KCl, NaCl, or the osmoprotectant GB. CFU were enumerated after 24 h of growth at 37°C. Data are means from three independent experiments performed in triplicate. Error bars represent one standard deviation from the mean. (D) Anaerobic viability of the WT or the *ypfP* mutant on medium buffered to a pH of 7 or 6. CFU were enumerated after 48 h of growth at 37°C. Data are means from three independent experiments performed in triplicate. Error bars represent one standard deviation from the mean. (E) The WT and the *ypfP* mutant were subcultured 1:100 in TSB, and the OD_600_ and pH were measured at the indicated time points. The data are averages from three independent experiments. Error bars represent one standard deviation from the mean. (B and C) Significance was determined using one-way ANOVA with multiple comparisons. ****, *P* < 0.0001; **, *P* <0.005.

Generation of the PMF is essential. PMF is a summation of the differences between intracellular and extracellular pH (ΔpH) and charge (Δψ) (36). S. aureus in which respiration is arrested demonstrates reduced membrane potential, and LTA has been proposed to maintain ion homeostasis (12, 22, 25). In keeping with these facts, we hypothesized that loss of ion homeostasis in anaerobically cultured *ypfP* mutant cells leads to further perturbation of the membrane potential, resulting in reduced proliferation during respiration arrest. To quantify the membrane potential of *ypfP* mutant cells, we used the fluorescent dye DiOC_2_ (diethyloxacarbocyanine iodide) (8, 37). Aerobically cultured WT and *ypfP* mutant cells generated similar membrane potentials. However, upon induction of anaerobiosis, the membrane potential generated by the *ypfP* mutant was significantly decreased (Fig. 4A). To discern between membrane potential and ΔpH, we used small-molecule inhibitors that specifically target each component of the PMF. First, we cultured the *ypfP* mutant in the presence of the proton ionophore nigericin or carbonyl cyanide 3-chlorophenyl hydrazine (CCCP) (38). However, no difference was observed in the *ypfP* mutant cell response to these ionophores under aerobic and anaerobic growth conditions (data not shown). Conversely, anaerobically cultured *ypfP* mutants are significantly more resistant to valinomycin, a potassium-specific ionophore that disrupts the membrane potential, than anaerobically grown WT cells (Fig. 4B) (39). We hypothesize that enhanced valinomycin resistance is consistent with a diminished membrane potential, as cells generating a depleted membrane potential would display great resistance to membrane potential-targeting ionophores. In keeping with this, we reasoned that increasing the abundance of extracellular charge by supplementing anaerobically cultured *ypfP* mutant cells with cations would overcome proliferation defects associated with respiration arrest and fermentative growth. To test this, we added KCl, NaCl, or H^+^ ions to anaerobically cultured *ypfP* mutants. Supplementation with 0.5 M KCl or 1 M NaCl rescued the anaerobic *ypfP* mutant viability (Fig. 4C). Similarly, decreasing the pH of the medium to 6.0 also rescued the viability defect of the *ypfP* mutant (Fig. 4D).

Another function attributed to LTA is maintenance of osmotic homeostasis, and addition of salts also increases the osmolarity of the medium. To discern between ion homeostasis and osmotic stress, we exposed anaerobically cultured *ypfP* mutants to the osmoprotectant glycine betaine (GB). However, addition of 1 mM or 0.5 M GB did not increase anaerobic viability (Fig. 4C and data not shown), indicating that perturbation of ion homeostasis is the major driver of the phenotype.

The *ypfP* mutant displayed an extended lag phase in anaerobic liquid culture but ultimately reached WT-like levels of growth (Fig. 2B). We hypothesized that the ability of the *ypfP* mutant to eventually reach an endpoint OD_600_ similar to that of the WT is due to acidification of the medium. To test this, the pH and OD_600_ of anaerobic WT and *ypfP* mutant cultures were monitored over time (Fig. 4E). Proliferation of the WT coincided with a drop in pH to ~4.9. Proliferation of the *ypfP* mutant was delayed but also coincided with a drop in pH to levels similar to those of the WT. In total, these data demonstrate that the defects in proliferation during respiration arrest associated with *ypfP* mutations are due to impaired ion homeostasis resulting in a reduced capacity to maintain the membrane potential.

### Mutations in the LTA biosynthetic pathway restore anaerobic viability in the *ypfP* mutant.

**FIG 5:**
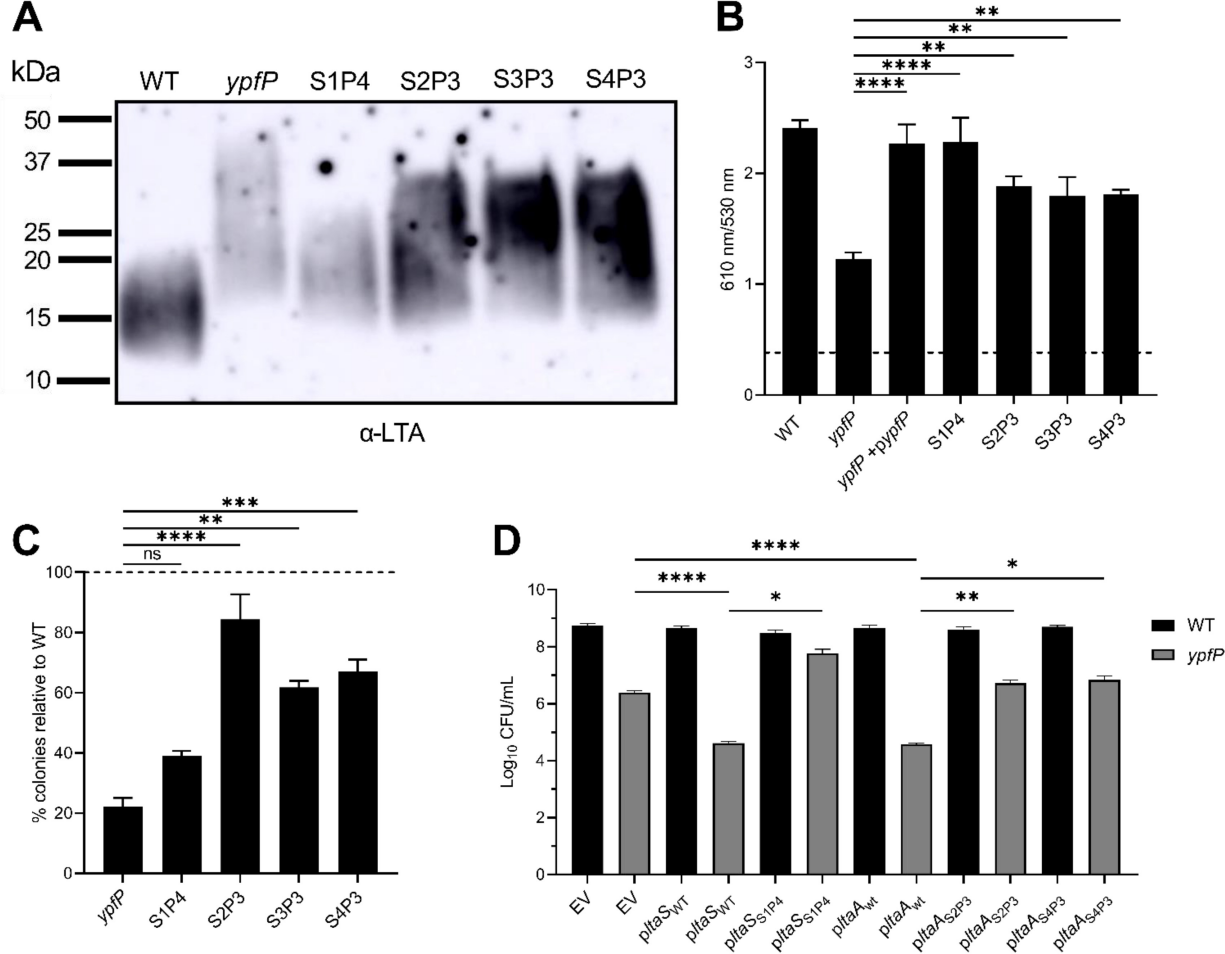
Passaged *ypfP* mutants harbor suppressor mutations that lead to phenotypic differences in membrane potential, gentamicin resistance and LTA. (A) Immunoblot of LTA isolated from cells grown aerobically to late exponential phase. Passaged *ypfP* mutants are named to reflect the lineage number and the passage at which they reached viability equal to that of the WT. For example, S1P4 represents passaged lineage 1, which reached WT-like viability after four passes under anaerobic conditions. (B) Anaerobic membrane potential was measured using the fluorescent dye DiOC_2_. Data are means from three independent experiments performed in technical triplicate. Error bars represent one standard deviation from the mean. Significance was determined using one-way ANOVA with multiple comparisons. ****, *P* < 0.0001; **, *P* < 0.01. (C) Percent gentamicin-resistant colonies relative to the WT (dotted line). Data represent 3 independent experiments. Error bars represent one standard deviation from the mean. Significance was determined using one-way ANOVA with multiple comparisons. ****, *P* < 0.0001; ***, *P* < 0.001; **, *P* < 0.001. (D) Strains were serially diluted, spot plated onto TSA, and incubated anaerobically and CFU were enumerated for the WT and the parental *ypfP* mutant complemented with empty pOS (EV), WT *ltaS* or *ltaA* (*ltaS*_WT_ and *ltaA*_WT_), mutated *ltaS* from S1P4 (*ltaS*_S1P4_), or mutated *ltaA* from S2P3 or S4P3 (*ltaA*_S2P3_ and *ltaA*_S4P3_). Data are means from 3 independent experiments. Error bars represent one standard deviation from the mean. Significance was determined using a two-tailed *t* test. ****, *P* < 0.0001; **, *P* < 0.01; *, *P* < 0.05.

To determine a potential mechanism for the anaerobic viability defect, the *ypfP* mutant was passaged anaerobically until a suppressor mutant that exhibited WT-like viability was isolated (Fig. S3). Passaging was repeated for four independently passaged lineages. Whole-genome sequencing revealed that the prophage-associated gene NWMN_1774 and genes in the LTA-biosynthetic pathway, *ltaS* and *ltaA*, were commonly mutated in the suppressor strains (Table S3). Suppressor lineage 1 passage 4 (S1P4) contained a mutation in *ltaS* (G39C). S2P3 and S3P3 harbored identical mutations in *ltaA* (K13N, N14R, F15L, and I16V). S4P3 also harbored a heavily mutated *ltaA* which shared many of the same mutations as S2P3 and S3P3 (K13N, N14R, and F15L), though two of them were unique (I16A and L17E). To determine the effect of these mutations, suppressor lineages were cultured aerobically to late exponential phase and their LTA was assessed via immunoblotting. Compared to the parental *ypfP* mutant, the suppressor mutants displayed lower-molecular-weight LTA, though it was still larger than that in the WT (Fig. 5A). Abundance of LTA in S1P4 was similar to that in the parental *ypfP* mutant (Fig. 5A, light band), while S2P3, S3P3, and S4P3 all exhibited WT-like LTA abundance (dark bands).

Due to the decreased membrane potential observed in anaerobically cultured *ypfP* cells and the fact that the suppressors produce altered LTA, we next determined whether the suppressor strains also restore anaerobic membrane potential. Notably, the four passaged lineages all exhibited anaerobic membrane potentials similar to that of the WT (Fig. 5B). The restored membrane potential of the passaged *ypfP* mutants suggests that producing WT-like LTA maintains membrane potential. The ability of the suppressor mutants to resist aminoglycosides was determined. S. aureus resists aminoglycosides by entering a fermentative state; therefore, we hypothesized that a *ypfP* suppressor mutant with anaerobic viability equal to that of the WT would have an improved ability to gain resistance to gentamicin. A partially restored ability to gain resistance to gentamicin was observed for S2P3, S3P3, and S4P3 but not S1P4 (Fig. 5C).

To determine whether mutations observed in *ltaS* and *ltaA* are responsible for the increased anaerobic viability of the suppressor lineages, the mutated alleles of these genes were cloned into pOS P*_lgt_* and used to complement the parental *ypfP* mutant (Fig. 5D). Overexpression of WT *ltaS* and *ltaA* in the parental *ypfP* mutant caused a further decrease in anaerobic viability compared to the empty vector. The mutated *ltaS* from S1P4 increased the anaerobic viability of the parental *ypfP* mutant to WT-like levels. S2P3 and S3P3 share identical *ltaA* mutations. Despite three independent suppressor lineages harboring *ltaA* mutations, there are only two unique mutated *ltaA* alleles, both of which partially restored the anaerobic viability of the parental *ypfP* mutant (Fig. 5D). Importantly, expression of WT alleles of *ltaS* or *ltaA* in the parental *ypfP* mutant background did not increase anaerobic viability, indicating that the increase in viability observed is not due to overexpression of the cloned genes but rather the specific mutations within the genes.

## DISCUSSION

Multiple respiration-arresting conditions impede proliferation of the S. aureus *ypfP* mutant, including exposure to the clinically relevant aminoglycoside antibiotics gentamicin and tobramycin, treatment with HQNO, and coculture with Pseudomonas aeruginosa. We posit that the reduced viability primarily stems from distorted LTA produced by the *ypfP* mutant, based on the observations that (i) alteration of the LTA produced by the *ypfP* mutant is exacerbated when the strain is cultured anaerobically, (ii) the anaerobic membrane potential generated in the *ypfP* mutant is significantly reduced, and (iii) the anaerobic viability defect is suppressed by supplementation with cations. The fact that LTA maintains ion homeostasis via binding of external cations to the negatively charged polyglycerol phosphates supports this model (12). If altered production of LTA causes decreased proliferation during respiration arrest, one would predict that inactivation of other LTA synthesis genes, such as *ltaA*, would elicit similar phenotypes. In fact, we observed that anaerobic viability of *ltaA* mutants was similar to that of the WT, indicating that defective proliferation during respiration arrest is specific to mutation of *ypfP* (Fig. 3C). On the surface, this result appears to conflict with a model implying a primary role for LTA, but there are significant differences between genetic inactivation of *ltaA* and *ypfP*. The first is that *ltaA* mutants retain production of Glc_2_-DAG, which accumulates in the membrane (17). This finding supports the notion that Glc_2_-DAG is protective in the *ltaA* mutant. However, another considerable difference between the mutants is the LTA they produce (Fig. 3D) (17). Notably, we demonstrate that whereas anaerobic growth further alters LTA produced by the *ypfP* mutant, it is relatively unperturbed in *ltaA* mutants (Fig. 3D). Additionally, our findings are similar to phenotypes observed when LTA production is completely ablated via *ltaS* mutation. *ltaS* mutant cells display severe viability defects that are suppressed by supplementation with high salt (20, 40, 41). The finding that anaerobic proliferation defects observed in *ypfP* mutants phenocopy *ltaS* inactivation, albeit to a lesser degree, lends additional support to the inference that disrupting LTA production is the primary driver of the respiration arrest-related growth defects. Therefore, defining the molecular nature of LTA produced in anaerobically cultured *ypfP* mutant cells will likely provide additional mechanistic details of ion homeostasis in S. aureus. For example, LTA GroP can be modified via addition of d-alanine residues to neutralize the negative charge and increase resistance to antimicrobial peptides (23). How this modification impacts ion homeostasis and membrane potential is uncertain but is an important consideration. Kiriukhin et al. found that LTA isolated from a *ypfP* mutant contained increased d-alanine compared to WT (19), while Fedtke et al. observed no difference in the d-alanylation status of LTA in *ypfP* mutant and WT cells (18). This conflicting evidence makes it unclear whether *ypfP* mutant LTA is differentially d-alanylated; however, further investigation is warranted for a more complete understanding of the role LTA and its modifications play in maintaining membrane potential. An alternative explanation for the observed phenotypes is that an unknown YpfP function also contributes. In keeping with this supposition, the YpfP homolog in Bacillus subtilis, UgtP, regulates cell division by inhibiting assembly of the FtsZ ring (42); however, a bacterial two-hybrid experiment failed to show an interaction between S. aureus YpfP and FtsZ proteins (43).

The essentiality of LTA has been established (21). It is interesting, then, that disruption of LTA would have such an impact on viability in cells with respiration arrest but have no discernible effect on respiratory growth. Our data suggest that LTA functions to maintain membrane potential; however, only the anaerobically cultured *ypfP* mutant displayed reduced membrane potential (Fig. 4A). A possible explanation is that during aerobic growth, the electron transport chain is functional and generates a proton gradient at the surface of the cell at a rate that is sufficiently high to maintain membrane potential despite the loss of cations to the surrounding medium. Conversely, cells in which respiration is arrested hydrolyze ATP in order to generate a proton gradient. Therefore, it is possible that anaerobically cultured cells spend considerable energy generating a proton gradient that is subsequently lost to the surrounding medium, ultimately depleting the ATP of the cell as it attempts to maintain the proton gradient. Such a dynamic is beyond the scope of this work but warrants further investigation.

Consistent with a model of LTA supporting anaerobic proliferation, all characterized *ypfP* suppressor lineages were shown to have lower-molecular-weight LTA than the parental *ypfP* mutant (Fig. 5A). Similar results were observed by Hesser et al., who found that *ltaS* mutations in a *ypfP* mutant background resulted in LTA more similar in length to that of the WT (44). While one of our suppressor lineages (S1P4) harbored a mutation in *ltaS*, three others (S2P3, S3P3, and S4P3) all had mutations in *ltaA*, which has not been previously reported to suppress the *ypfP* mutant phenotype. The lower-molecular-weight LTA likely restored the ability of the cell to maintain membrane potential (Fig. 5B), highlighting the importance of LTA length in this phenotype. However, when challenged with gentamicin, S1P4, which exhibited decreased LTA abundance, did not regain the capacity to resist the aminoglycoside. In addition to entering a fermentative state to gain gentamicin resistance, it may be possible that the relative abundance of LTA also plays an important role. In keeping with this, S2P3, S3P3, and S4P3 were able to generate gentamicin resistance, and all exhibited relatively greater LTA abundance than the parental *ypfP* mutant (Fig. 5A, dark bands). Conversely, S1P4 displayed LTA abundance more similar to that of the parental *ypfP* mutant (Fig. 5A, light band). LTA has been implicated in facilitating resistance to cationic antimicrobial peptides, likely by regulating the cell surface charge via d-alanylation (23, 45), which has been known to affect the binding of aminoglycosides to teichoic acids (46). Furthermore, abundance of LTA has been shown to promote antibiotic resistance, as S. aureus cells with reduced cell-associated LTA were shown to be more sensitive to oxacillin (47). Therefore, it is likely that both the length and abundance of LTA are important factors that coordinate the necessary mechanisms needed for gentamicin resistance.

Our results show that the LTA glycolipid anchor is required for maximal metabolic versatility of S. aureus. Cells deficient for its production demonstrate severe proliferation defects during respiration arrest, likely due to a loss of membrane potential. We show that cells with LTA more similar to the WT in abundance and size are able to restore fermentative viability and more readily resist aminoglycoside treatment. Therefore, LTA continues to be a promising target for the treatment of recalcitrant S. aureus infections, and its disruption could enable the use of antibiotics that were previously ineffective.

## MATERIALS AND METHODS

### Bacterial strains and growth conditions used in this study.

Bacterial strains used in this study are described in Table S1. All strains are derivatives of S. aureus isolate Newman (NWMN) (48). S. aureus was routinely cultured in tryptic soy broth (TSB) or TSA. The *ypfP* and *ltaA* transposon insertion mutants were obtained from the Nebraska Transposon Mutant Library and transduced into the respective backgrounds via a phage ϕ85 transduction protocol as previously described (49). Antibiotic selection of erythromycin cassette-containing resistant recipient cells was achieved with 10 μg mL^−1^ erythromycin. Transposon mutants were verified by PCR using primers described in Table S2. δ-Aminolevulinic acid (ALA) was supplemented at 75 μg mL^−1^. The Δ*gtrR* mutant was created as described previously (8). The deletion was confirmed via PCR. Individual Δ*gtrR* mutants were checked for hemolytic activity on blood agar (Thermo Fisher Scientific). Mutants that retained hemolytic activity were tested for second-site mutations in the *sae* locus via Sanger sequencing of the *saeS* gene. Tobramycin was used at 5 μg mL^−1^, and gentamicin was used at 5 μg mL^−1^ or 2 μg mL^−1^, as indicated. Sodium chloride or potassium chloride was added to TSA at the indicated concentrations and mixed prior to sterilization. The pOS-*ypfP* plasmid was created using primers listed in Table S2, the restriction enzymes XhoI and BamHI, and traditional ligation cloning methods. Plasmids pOS-*ltaA*, pOS-*ypfPltaA*, pOS-*ltaS*, and pOS-*ltaS*_S1P4_ were created using the Gibson Assembly cloning kit (New England Biolabs). Plasmids pOS-*ltaA*_S2P3_ and pOS-*ltaA*_S4P3_ containing the mutated *ltaA* genes seen in S2P3 and S4P3 were generated via divergent PCR using pOS-*ltaA*_WT_ as a template with the primers indicated in Table S2. Linear PCR products were then recircularized before transformation. After construction, all plasmids were transformed into Escherichia coli DH5α. Selection of plasmid-containing clones was achieved using 100 μg mL^−1^ ampicillin and validated via sequencing performed at the Michigan State University Research Technology and Support Facility using primers pOS seq F and pOS seq R (Table S2) or Plasmidsaurus. Plasmids were then electroporated into S. aureus strain RN4220 as an intermediate host before transformation into S. aureus strain NWMN. Selection of plasmid-containing clones in S. aureus and maintenance of plasmid-containing strains was achieved using 10 μg mL^−1^ chloramphenicol, unless otherwise specified. Overnight cultures were normalized to an OD_600_ of 1.0 and then diluted 1:100 into TSB for growth curve analysis. Flasks were incubated at 37°C shaking at 225 rpm for aerobic growth kinetics, and polystyrene tubes were incubated without shaking at 37°C in a Coy anaerobic chamber. Three aliquots of 200 μL were pipetted into a 96-well plate, and the OD_600_ was measured at 2-h intervals for aerobic cultures or 3-h intervals for anaerobic cultures.

### Determination of percent aminoglycoside resistance.

Overnight cultures of WT and *ypfP* mutant cells were normalized to an OD_600_ of 0.1 in phosphate-buffered saline (PBS), and 100 μL was plated onto TSA containing 5 μg mL^−1^ gentamicin or tobramycin. Plates were incubated at 37°C and colonies were enumerate after 24 h. Percent relative resistance was determined by dividing the number of colonies of the mutant strain by the number of colonies of the WT. Values were then plotted, and the WT value was set to 100%.

### P. aeruginosa coculture and HQNO Kirby-Bauer assays.

Overnight cultures of WT or *ypfP* mutant cells were plated onto 20-mL TSA plates using sterile cotton swabs. Following plating, 2 μL of an overnight culture of P. aeruginosa PAO1 was spotted in the middle of the plate. Similarly, 20-mL TSA plates containing the WT or the *ypfP* mutant were prepared, and a sterile Whatman disk containing 10 μL of 1 mg mL^−1^ HQNO or pyocyanin (Cayman Chemical) was placed in the center of the plate. Plates were incubated at 37°C overnight. The zone of inhibition and the diameter of the P. aeruginosa colony were measured using an electronic caliper (Pittsburgh 6-in. composite digital caliper).

### Aerobic and anaerobic CFU enumeration.

Overnight cultures were normalized to an OD_600_ of 1.0 in PBS. The normalized culture was serially diluted in PBS in a 96-well plate. Ten microliters of each dilution was spotted onto TSA. Plates were incubated at 37°C aerobically or in an anaerobic chamber (Coy). Colonies were enumerated after 24 h (aerobic) or 48 h (anaerobic), and the number of CFU per milliliter was calculated. Each experiment was performed in biological triplicate, with three technical replicates per biological replicate. The three log values were then plotted using GraphPad Prism.

### LTA Western blot analysis.

For aerobic versus anaerobic analysis of LTA, overnight cultures were normalized to the OD_600_ of WT cells, subcultured 1:1,000 in TSB, and grown with shaking at 225 rpm at 37°C for 15 h aerobically or anaerobically. After 15 h of growth, the cultures were normalized to the WT OD_600_ in 1-mL aliquots in 2-mL screw-cap tubes containing 0.5 mL 0.1-mm zircon beads. The cells were subjected to bead beating for 45 s on a Mini-Beadbeater 16 (BioSpec). The tubes were then centrifuged at 300 × *g* for 1 min, and the supernatant was decanted into a fresh sterile 1.5-mL Eppendorf tube. The supernatant was centrifuged at 13,300 × *g* for 15 min to sediment membranes and LTA. The pellet was resuspended in 80 μL of PBS. Twenty microliters of 5× SDS sample buffer was added to the resuspended pellet. Twenty microliters of the mixture was loaded into a 15% poly-bis-acrylamide gel. Samples were run for 10 min at 50 V and then ~2 h at 100 V or until the dye front reached the bottom of the gel. Samples were transferred to polyvinylidene fluoride (PVDF) at 4°C at 100 V for 1 h. The PVDF membrane was blocked using PBST (1× PBS plus 0.05% [vol/vol] Tween 20 plus 5% milk) blocking buffer for 1 h, washed twice in fresh blocking buffer for 10 min each time, and exposed to blocking buffer supplemented with a 1:1,000 dilution of primary anti-LTA (polyglycerol-specific) mouse antibody (Hycult Biotechnology). After 1 h, the membrane was washed twice in blocking buffer and exposed to blocking buffer containing 1:5,000 horseradish peroxidase-conjugated secondary anti-mouse immunoglobulin goat antibody (Millipore) for an additional hour. Enhanced chemiluminescence (ECL) western blotting substrate (Promega) and film were used to detect immunoreactive LTA.

For the analysis of LTA from *ypfP* suppressor lineages, overnight cultures were started from a single colony and grown in TSB overnight at 37°C. Cultures were back diluted 1:100 in fresh TSB and grown aerobically to an OD_600_ equal to 0.7. A 1-mL aliquot was pelleted and resuspended in 45 μL TSM (50 mM Tris, 500 mM sucrose, 10 mM MgCl_2_; pH 7.5). The cell wall was digested by adding 5 μL of 2 mg/mL lysostaphin and incubating at 37°C for 30 min. After incubation, 50 μL of 2× SDS-PAGE loading buffer was added, and the sample was incubated at 95°C for 30 min. The sample was then centrifuged at 13,000 × *g* for 2 min and the supernatant was collected. A 1-μL volume of proteinase K (Thermo Fisher Scientific) was added to the sample and incubated at 50°C for 2 h. For each sample, 20 μL was loaded into a 4-to-20% poly-bis-acrylamide gel (Bio-Rad). Samples were run for 30 min at 90 V and then 45 min at 150 V. The gel was then transferred and stained as described above.

### Measurement of membrane potential.

The electrochemical potential of the cells was determined using the fluorescent dye DiOC_2_ (Millipore-Sigma). Cultures were grown to exponential phase aerobically (OD_600_ equal to 0.6) or anaerobically (OD_600_ equal to 0.3). All aerobic cultures reached exponential phase after approximately 3.5 h of growth. Anaerobic cultures reached exponential phase at approximately 5 h (WT), 8 h (S1P4, S2P3, S3P3, and S4P3), and 14 h (*ypfP* mutant). Culture volumes of 4 mL were pelleted and resuspended in an equal volume of PBS. Three 1-mL aliquots of the resuspension were made. One aliquot received no treatment, one received 10 μL of 500 μM CCCP as a negative control and 10 μL of 3 mM DiOC_2_, and the last aliquot received 10 μL of DiOC_2_. The tubes were incubated in the dark for 25 min at room temperature. After incubation, three 200-μL samples from each tube were pipetted into three wells of a black-walled 96-well plate. Fluorescence emission was measured at 530 nm and 610 nm from an excitation at 488 nm using a Synergy H1 plate reader (Biotek). Autofluorescence values from the untreated samples were subtracted from both the CCCP-treated and non-CCCP-treated samples prior to calculation of the ratio. The data represents the 610-nm fluorescence divided by the 530-nm fluorescence.

### Optical density and pH measurements of anaerobic liquid cultures.

Overnight 5-mL cultures of the WT and *ypfP* mutant were started from single colonies and grown at 37°C with shaking. Cells were pelleted, washed in PBS, and normalized to an OD_600_ of 1.0. Cultures (100 mL) were inoculated to a starting OD_600_ of 0.01 and grown anaerobically at 37°C in a Coy anaerobic chamber. Ten-milliliter volumes of the WT and *ypfP* cultures were collected at 0-, 2-, 4-, 6-, 8-, 12- and 24-h time points. A 1-mL volume of each time point sample was used to measure OD_600_ with a Genesys 140 Visible spectrophotometer (Thermo Fisher Scientific). The remaining 9 mL of sample was pelleted, and the supernatant was filtered. The pH of the sterile supernatant was measured using a FiveEasy Plus FEP20 pH meter (Mettler Toledo).

### Antimicrobial activity assays of PMF-targeting inhibitors.

CCCP, valinomycin, and nigericin were solubilized in dimethyl sulfoxide (DMSO). The compounds were serially diluted 2-fold in 96-well plates prior to inoculation. The plates were incubated at 37°C for 16 h, the wells were then resuspended via pipetting, and the OD was measured at 600 nm. The values for the untreated control wells were averaged, and this value was used to represent 100% growth. The values for the rest of the wells were averaged and compared to that 100% value. Experiments were performed in at least biological triplicate with three technical replicates per biological replicate unless otherwise indicated. The results for technical replicates were averaged for each biological replicate, and the values for biological replicates were graphed using GraphPad Prism. The regression line for nonlinear fit was used to determine the best fit line.

### Fluorescence microscopy.

S. aureus cultures were grown overnight at 37°C in TSB and subsequently diluted 1:10 in fresh medium. Cultures were then grown to mid-log phase (OD_600_ equal to 0.5) at 37°C and treated with 2 μg mL^−1^ gentamicin, where indicated, for a period of 2 h. Following the growth period, 1-mL aliquots of the cultures were washed in 1× PBS, and then cell pellets were resuspended in 100 μL of 1× PBS. Cells were stained with 1 μg mL^−1^ FM4-64 in order to visualize the cell membrane. Five microliters of sample was transferred to a glass-bottom culture dish (MatTek) and then covered with a 1% agarose pad. All imaging was conducted at room temperature using a DeltaVision Core microscope system (Applied Precision/GE Healthcare) equipped with an environmental chamber. All images were captured using a Photometrics Coolsnap HQ2 camera, and data were deconvolved using SoftWorx software supplied by the microscope manufacturer.

### Isolation of *ypfP* suppressor mutants and SNP analysis.

From individual colonies, overnight cultures of WT and the *ypfP* mutant were incubated at 37°C with shaking. Cultures were normalized to an OD_600_ of 1.0 in TSB. Normalized growth was serially diluted in PBS in a 96-well plate, and 10 μL of each dilution was spotted on two TSA plates, one of which was incubated aerobically (as a control) at 37°C and the other anaerobically at 37°C. Large, individual *ypfP* mutant colonies were selected from the anerobic plate and cultured aerobically in 5 mL TSB at 37°C with shaking. The overnight culture was then serially diluted again and spot plated as described above, along with a newly cultured WT as a reference for viability. This process was repeated until a *ypfP* suppressor mutant was obtained that had viability equal to that of the WT and is visually summarized in Fig. S3.

Genomic DNA was isolated from 5-mL overnight cultures started from individual colonies grown aerobically at 37°C with shaking. The culture was pelleted and resuspended in 485 μL TSM. A volume of 15 μL 2 mg/mL lysostaphin was added to the resuspension and incubated at 37°C for 30 min. The samples were then pelleted, and the Wizard genomic DNA purification kit (Promega) was used to isolate the genomic DNA. Whole-genome sequencing was performed via Illumina sequencing at the Microbial Genome Sequencing Center. Sequence read processing, alignment, and single nucleotide polymorphism (SNP) analysis were performed using Geneious Prime version 2022.0.2. The genomic sequence of S. aureus strain Newman (GenBank accession number AP009351.1) was used as a reference genome for all genomic analysis.
