# Supplementary material for: Crucial Role for Lipoteichoic Acid Assembly in the Metabolic Versatility and Antibiotic Resistance of Staphylococcus aureus
Source: Infect Immun. 2023 Jun 22;91(7):e00550-22. doi: 10.1128/iai.00550-22 (PMC10353449; doi:10.1128/iai.00550-22)
Supplement: Supplemental file 1 — Tables S1 to S3 and Fig. S1 to S3. Download iai.00550-22-s0001.docx, DOCX file, 1.1 MB [file iai.00550-22-s0001.docx]

**SUPPLEMENTAL INFORMATION**

**Table S1. Bacterial strains used in this study.**

|  | | |
| --- | --- | --- |
| **Name** | **Relevant genotype and phenotype** | **Source** |
| ***Staphylococcus*** ***aureus*** | | |
| RN4220 | Restriction deficient, methylation proficient | 65 |
| Newman (NWMN) | WT | 57 |
| *ypfP* | *ypfP::erm* | this study |
| *ltaA* | *ltaA::erm* | this study |
| ∆*gtrR* | in-frame deletion, heme synthesis deficient small colony variant | this study |
| ∆*gtrR* *ypfP* | heme synthesis deficient small colony variant *ypfP::erm* | this study |
| S1P4 | *ypfP::errm* anaerobic suppressor (Table S3) | this study |
| S2P3 | *ypfP::erm* anaerobic suppressor (Table S3) | this study |
| S3P3 | *ypfP::erm* anaerobic suppressor (Table S3) | this study |
| S4P3 | *ypfP::erm* anaerobic suppressor (Table S3) | this study |
|  | | |
| ***Pseudomonas aeruginosa*** | | |
| PAO1 | WT | 34 |

| **Table S2. Primers used in this study.** | | |
| --- | --- | --- |
| **Primer name** | **Sequence** | **Source** |
| NE Buster^a^ | GCTTTTTCTAAATGTTTTTTAAGTAAATCAAGTAAC | 48 |
| NE Martn-ermR^a^ | AAACTGAATTTTAGTAAACAGTTGACGATATTC | 48 |
| *ltaA* Tn seq.^a^ | GCGCTCGAGATGGAAAGGTTCCTTTATATGC | this study |
| *ypfP* Tn seq.^a^ | GCGTCATTGAGCACGATTTATT | this study |
| *ltaA*-F Gibson^a^ | ACAATTGAGGTGAACATATGGAAAGGTTCCTTTATATG | this study |
| *ltaA*-R Gibson^a^ | CTACCCCCTTGTTTGGATCCTTACTTAGCTTTTTCTCTATTTAC | this study |
| pOS seq. F | TTAGCTTTTCAATGTAGATTGG | this study |
| pOS seq. R | ATTACGCCAAGCTAGCTTGG | this study |
| *ypfP*-F pOS^a^ | GCGCTCGAGATGGTTACTCAAAATAAAAAGATAT | this study |
| *ypfP*-R pOS^a^ | GCGGGATCCTTATTTAACGAAGAATCTTGAATA | this study |
| *ltaA-*mut^b^ | ATGATTAGCGTAATTATTTAACGAAGAATCTTGC | this study |
| *ltaA*-S2P3^b^ | AACAGGTTGGTTTTAATGCTTATTATCTTATTTTTAATGGAATTTGCGAG | this study |
| *ltaA*-S4P3^b^ | AACAGGTTGGCTGAAATGCTTATTATCTTATTTTTAATGGAATTTGCGAG | this study |
| *ltaS-*F Gibson^a^ | ACAATTGAGGTGAACATATGAGTTCACAAAAAAAGAAAATTAGTC | this study |
| *ltaS*-R Gibson^a^ | ACTACCCCCTTGTTTGGATCTTATTTTTTAGAGTTTGCTTTAGG | this study |
| pOS-F Gibson^c^ | GATCCAAACAAGGGGGTAGTGT | this study |
| pOS-R Gibson^c^ | CATATGTTCACCTCAATTGTATTTATCCCTAC | this study |
| ^a^Indicates primers used on genomic DNA to amplify its respective target sequence  ^b^Indicates the pOS-*ltaA* complementation plasmid was used as the template sequence  ^c^Primers used to amplify/linearize pOS for downstream use in Gibson assembly for cloning of *ltaS* | | |

| **Table S3. Mutational profiles of *ypfP* suppressor mutants.** | | | | | |
| --- | --- | --- | --- | --- | --- |
| **S1P4** | | | | | |
| **Gene** | **Name** | **Product^a^** | **TIGRFAM role^b^** | **Mutation type** | **Protein effect** |
| NWMN_0300 | - | Hypothetical protein | Unknown | Missense | G159V |
| NWMN_0407 | *lpl4* | Tandem-type lipoprotein | *Staphylococcus* tandem lipoproteins | Deletion, Insertion | Frame shift, Frame shift |
| NWMN_0687 | *ltaS* | Lipoteichoic acid synthase | Choline-sulfatase, Frataxin | Missense | G39C |
| NWMN_1774 | - | Hypothetical protein | MutS2 family protein | Missense | L14I, T15R |
| **S2P3** | | | | | |
| NWMN_0886 | *ltaA* | Proton coupled antiporter flippase | Miltidrug resisatance protein, H+ Antiporter protein | Missense | K13N, N14R, F15L, I16V |
| NWMN_1774 | - | Hypothetical protein | MutS2 family protein | Missense | L14I, T15R |
| **S3P3** | | | | | |
| NWMN_0309 | - | Phage N-acetylglucosamidase | Flagellar rod assembly protein/muramidase FlgJ | Missense | S478A |
| NWMN_0886 | *ltaA* | Proton coupled antiporter flippase | Miltidrug resisatance protein, H+ Antiporter protein | Missense | K13N, N14R, F15L, I16V |
| NWMN_1622 | *tyrS* | Tyrosine tRNA ligase | tRNA aminoacylation | Missense | L9F |
| NWMN_1774 | - | Hypothetical protein | MutS2 family protein | Missense | L14I, T15R |
| NWMN_2337 | - | Amino acid permease | GABA permease, Amino acid permease | Missense | M161L |
| **S4P3** | | | | | |
| NWMN_0486 | *mcsB* | ATP:guanido phosphotransferase | Unknown | Missense | I84L |
| NWMN_0886 | *ltaA* | Proton coupled antiporter flippase | Miltidrug resisatance protein, H+ Antiporter protein | Missense | K13N, N14R, F15L, I16A, L17E |
| NWMN_1774 | - | Hypothetical protein | MutS2 family protein | Missense | L14I, T15R |
| ^a^Products for each gene were obtained from AureoWiki (63).  ^b^The role of the protein product of each gene was predicted by the TIGRFAM database (64). In the case of multiple roles being predicted, the top two highest scoring roles were shown. Protein products without a predicted role were listed as unknown. | | | | | |

**FIGURE S1**


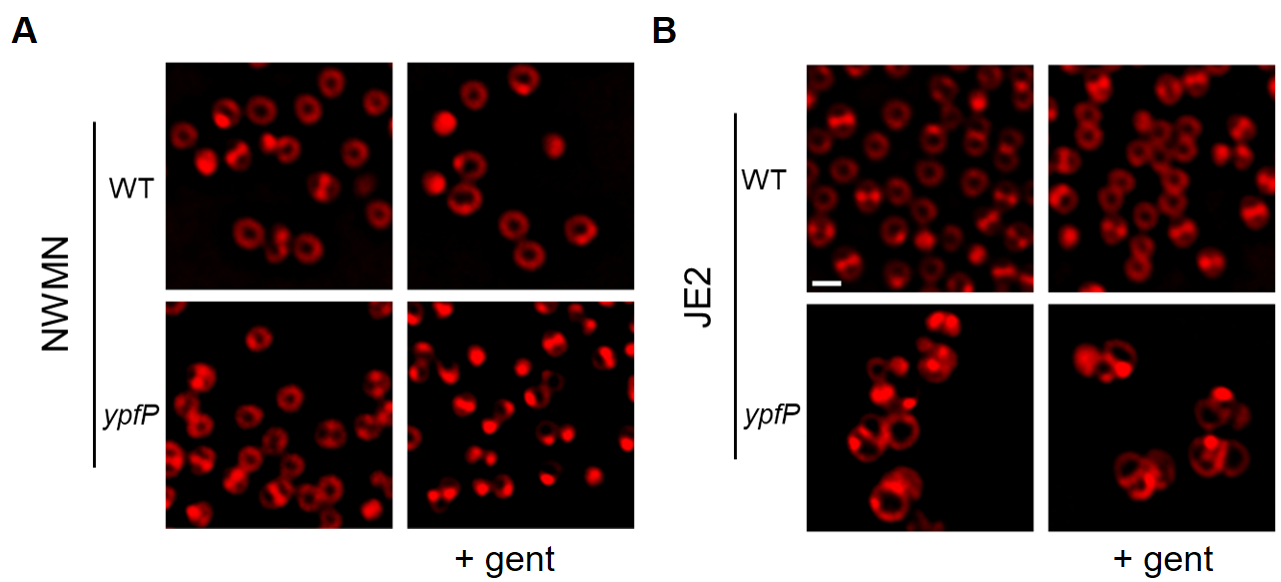


**Figure S1. Exposure to gentamicin causes increased morphological defects and membrane clumping in the *ypfP* mutant.** Fluorescence micrographs of FM4-64 (red) stained WT and *ypfP* mutant cells following treatment without (left panels) or with 2 µg mL^-1^ gentamicin (right panels). Scale bar, 1 µm.

**FIGURE S2**


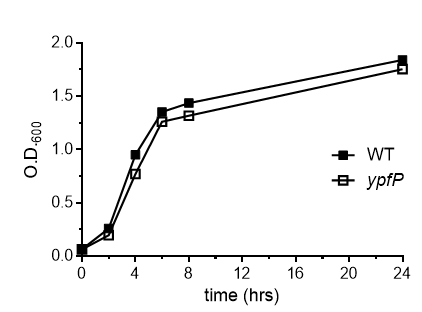


**Figure S2. *S. aureus* *ypfP* mutant cells demonstrate wild type-like aerobic growth kinetics.** WT and *ypfP* were sub-cultured 1:100 from an overnight culture and grown anaerobically at 37° C. Growth was measured at indicated time points by monitoring optical density at 600 nm (OD_600_). The experiment was performed in triplicate. Error bars represent one standard deviation from the mean.

**FIGURE S3**

**
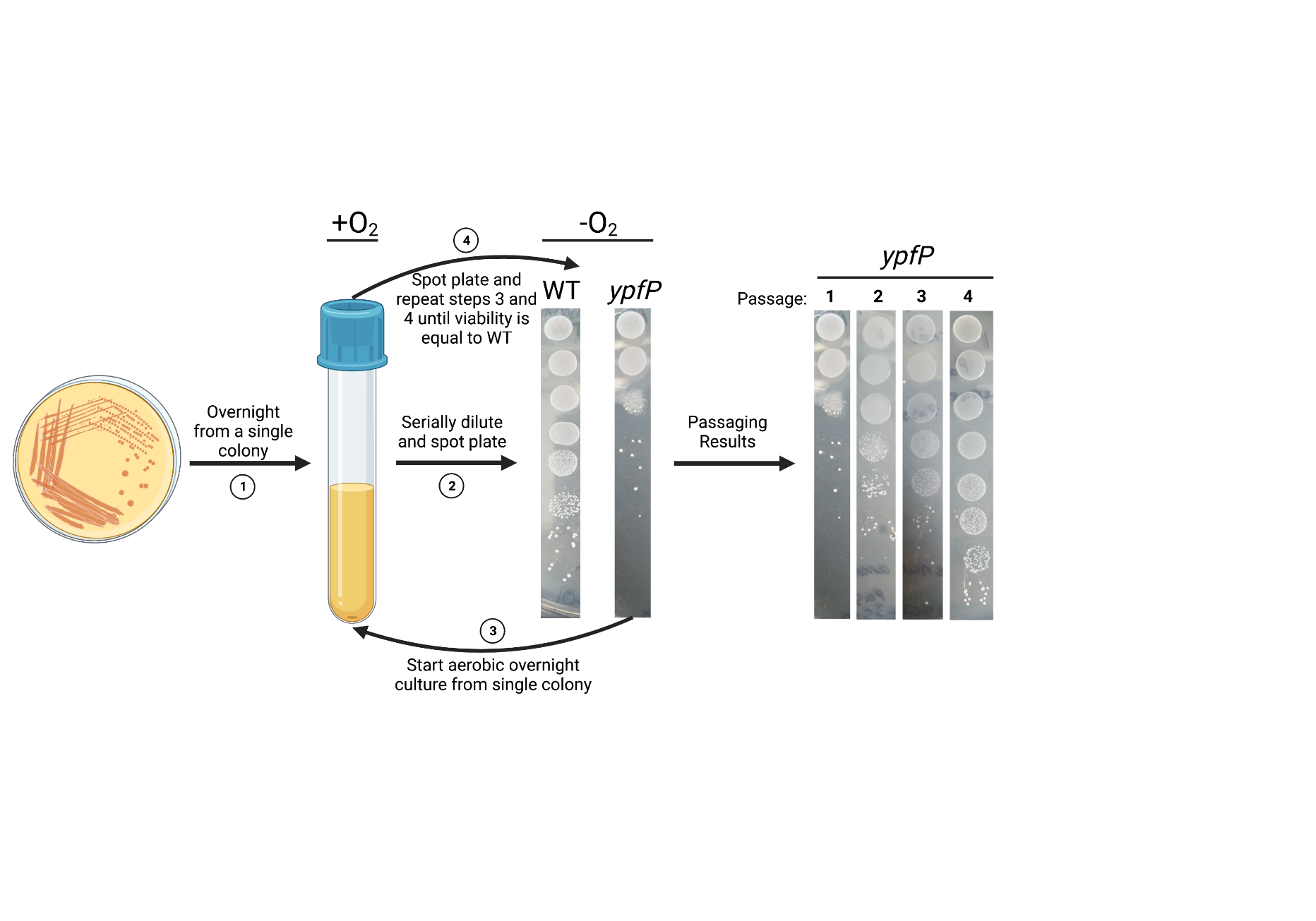
**

**Figure S3. An illustration of the method used to isolate *ypfP* suppressor mutants.** For more detailed information see methods. 1) An overnight culture was started from an individual colony. 2) The overnight culture was serially diluted, spot platted, and incubated in anaerobic conditions. 3) An individual colony from the *ypfP* spot plate was used to start an aerobic overnight culture. At this point, one pass has been completed. 4) The passaged *ypfP* mutant was serially diluted and spot plated again and repeated until viability was equal to the WT. Image created using BioRender.com.
